# ARHGAP44-mediated regulation of the p53/C-myc/Cyclin D1 pathway in modulating the malignant biological behavior of osteosarcoma cells

**DOI:** 10.1186/s13018-023-04406-z

**Published:** 2023-11-29

**Authors:** Shizhe Li, Jiancheng Xue, He Zhang, Guanning Shang

**Affiliations:** 1 Department of Orthopedics, The First Affiliated Hospital of Jinzhou Medical University, Jinzhou, Liaoning China; 2 Department of Orthopedics, Shengjing Hospital of China Medical University, Shenyang, Liaoning China; 3 Medical Research Center, Shengjing Hospital of China Medical University, Shenyang, Liaoning China

**Keywords:** *ARHGAP44*, Osteosarcoma, *p53*, Proliferation, Invasion, Migration

## Abstract

**Objective:**

Osteosarcoma is a rare primary malignant tumor of the bone characterized by poor survival rates, owing to its unclear pathogenesis. Rho GTPase-activating protein 44 (*ARHGAP44*), which belongs to the Rho GTPase-activating protein family, has promising applications in the targeted therapy of tumors. Therefore, this study aimed to investigate the biological function of *ARHGAP44* in osteosarcoma and its possible application as a therapeutic target.

**Methods:**

The expression level of *ARHGAP44* in osteosarcoma and its relationship with tumor prognosis were detected using Gene Expression Omnibus database analysis and immunohistochemical staining of clinical specimens. The cell model of *ARHGAP44* knockdown was constructed, and the effects of this gene on the malignant biological behavior of osteosarcoma cells were investigated using CCK-8, clone formation, transwell invasion, wound healing, and flow cytometry assays. Western blotting was performed to detect the expression of *ARHGAP44*, *p53*, *C-myc*, and *Cyclin D1* in osteosarcoma.

**Results:**

Biogenic analysis showed that *ARHGAP44* was highly expressed in osteosarcoma. This result was associated with poor tumor prognosis and negatively correlated with the expression of the tumor suppressor gene *p53*. Immunohistochemistry and western blotting revealed significantly upregulated expression of *ARHGAP44* in osteosarcoma tissues. Additionally, Kaplan–Meier analysis of clinical specimens suggested that *ARHGAP44* was negatively correlated with tumor prognosis. CCK-8, clone formation, transwell invasion, wound healing, and flow cytometry assays showed that downregulation of *ARHGAP44* expression significantly reduced the malignant biological behavior of osteosarcoma cells. Furthermore, western blotting showed that the expression level of *p53* in osteosarcoma cells was significantly increased after the downregulation of *ARHGAP44* expression, whereas the expression of *C-myc* and *Cyclin D1* was significantly decreased compared with that in the control group.

**Conclusion:**

*ARHGAP44* was highly expressed in osteosarcoma and was negatively correlated with its prognosis. The downregulation of *ARHGAP44* expression reduced the malignant biological behavior of osteosarcoma cells. These findings suggest that the downregulation of *ARHGAP44* expression inhibits the malignant progression of osteosarcoma by regulating the *p53*/*C-myc*/*Cyclin D1* pathway, demonstrating the potential of *ARHGAP44* as a therapeutic target for osteosarcoma.

## Introduction

Osteosarcoma is a rare primary malignant bone tumor that occurs in children and adolescents [1]. The current treatment strategy for osteosarcoma is limb-sparing surgery combined with adjuvant chemotherapy and targeted therapy, which can increase the 5-year overall survival rate of patients with non-metastatic recurrent osteosarcoma to approximately 70% [2]. However, the prognosis of osteosarcoma often deteriorates following recurrence or distant metastasis. In addition, the inability to undergo surgical resection or resistance to chemotherapy often lead to a lack of effective treatment for metastatic or recurrent cases [3]. Modern medical technology and molecular biology have enabled the rapid development of targeted anticancer drugs [4]. In addition, targeted therapy for osteosarcoma has broader research prospects because this tumor involves more targets and signaling pathways compared to benign bone tumors [5].

Rho GTPase-activating protein 44 (*ARHGAP44*) belongs to the Rho GTPase-activating protein (*RhoGAP*) family, also known as Rich2 [6]. Loss of *RhoGAP* activity often leads to uncontrolled GTPase activity, which consequently affects tumor progression. *RhoGAP* factors can also affect tumors by regulating the cell cycle, including non-small cell lung and colorectal cancers [7]. The role of *ARHGAP44* in tumors is more controversial than that of other *ARHGAP* members. The upregulation of *ARHGAP44* expression is associated with aberrant breast follicular morphology [8]. Another study showed the cancer-suppressive effect of upregulated *ARHGAP44* expression in hepatocellular carcinoma [9]. Hu et al. [10] suggested that the overexpression of this gene promoted osteosarcoma metastasis and was associated with poor prognosis through gene pool enrichment analysis. However, no further experiments were performed to elucidate the mechanism. In addition, Xu et al. [11] found that mutant *p53* inhibits the transcription of *ARHGAP44*, thereby affecting the spread and migration of tumor cells.

In this study, we used multiple biological techniques at several levels, including clinical specimens, proteins, and molecules, to elucidate the impact of *ARHGAP44* on the mechanism underlying osteosarcoma development and its participation in the detailed processes of the malignant biological behavior of osteosarcoma cells through the *p53* signaling pathway.

## Materials and methods

### Bioinformatic analysis

The mRNA chips GSE14359 [12] and GSE39058 [13] were downloaded from the Gene Expression Omnibus (GEO) database (http://www.ncbi.nlm). GSE14359 contained 18 tumor tissue and two normal samples from patients with osteosarcoma but no survival information was obtained (Table 1). In contrast, GSE39058 included miRNA data and survival information of 65 patients (including 65 biopsy specimens and 26 pairs of surgically resected specimens) (Table 2). GSE14359 was processed using Illumina Human software to analyze expression differences. Survival of the high and low expression groups in the GSE39058 dataset was determined, and KM survival curves were plotted according to the Kaplan–Meier method, in which the high and low expression groups were classified according to the expression level of *ARHGAP44* from high to low at the median. RNA-seq data for osteosarcoma (Level 3) were obtained from The Cancer Genome Atlas (TCGA) database (https://portal.gdc.com). Genes contained in the corresponding *p53* pathway were collected. The correlation between *ARHGAP44* and potential targets was analyzed using the R software GSVA package and selecting the parameter method = 'ssgsea.'

**Table 1:** Clinical information of patients with osteosarcoma GSE14359

Clinical information	Number (n = 20)
Type	
Non-neoplastic primary osteoblast cells	2
Conventional osteosarcoma tissue	10
Osteosarcoma lung metastasis tissue	8
Age	
Median	23
Range	7–74

**Table 2:** Clinical information of patients with osteosarcoma GSE39058

Clinical information	Number (n = 65)
Age	
Median	12
Range	3–76
Sex	
Male	30 (46%)
Female	35 (54%)
Metastases at diagnosis	
Yes	11 (17%)
No	54 (83%)
Tumor status	
Recurrence	23 (35%)
Death	14 (22%)

### Immunohistochemical staining

Tissue samples were collected from 21 patients with osteosarcoma at the Department of Orthopedics, Shengjing Hospital of China Medical University (Table 3). Inclusion criteria: (1) Patients diagnosed with conventional osteosarcoma through postoperative pathology; (2) patients whose tumors had been radiologically evaluated and could be surgically removed; and (3) patients who did not receive radiotherapy or chemotherapy. Exclusion criteria: (1) patients younger than 6 years old; (2) patients with other serious diseases; and (3) patients with incomplete history and imaging data. All study protocols involving patients were conducted in accordance with the declaration of Helsinki and provided informed consent prior to the initiation of experiments.

**Table 3:** Immunohistochemistry patient clinical information

Clinical information	Number (n = 21)
Age	
Median	16
Range	8–73
Sex	
Male	11 (52%)
Female	10 (48%)
Metastases at diagnosis	
Yes	3 (14%)
No	18 (86%)
Tumor status	
Death	6 (29%)

Patients underwent excision biopsy to obtain paracancerous tissue from bone-like tissue in the 1–3 cm region of the gross visible margin of the tumor. Tissue samples were embedded in paraffin, cut into 5 μm paraffin sections, dewaxed, and repaired by microwaving for 10 min with a citric acid solution. Endogenous peroxidase blockers and serum blocking solution were added to the slides for 40 min, followed by incubation with *ARHGAP44* antibody (1:150; Atlas Antibodies, Bromma, Sweden) at 4 °C overnight. DAB color development, re-dyeing, and dewatering sealing were performed on the second day. Positive results were analyzed using a semi-quantitative scoring method, where the percentage of positive cell counts (0–100%) was combined with the intensity of staining (0 = negative, 1 = weakly positive, 3 = moderately positive, 4 = strongly positive), and the final data were the product of the two indices. KM prognostic analysis was also performed on the collected clinical specimens based on immunohistochemical scores to assess their relationship with tumor prognosis.

### Cell culture and siRNA transfection

Human osteosarcoma cell lines MG63 and HOS and the osteoblastic cell line hFOB1.19 were purchased from Procell Life Science and Technology Co. Ltd. (Wuhan, China). The MG63 and HOS cells were cultured in minimum essential medium (MEM) (Procell, Wuhan, China) containing 10% fetal bovine serum (FBS), whereas the hFOB1.19 cells were cultured in Dulbecco’s modified Eagle’s medium (DMEM) (Procell) containing G418 (Procell) and 10% FBS. They were all placed in an incubator at 37 °C and 5% CO_2_ to enable cell growth.

Cells were transfected using the Lipofectamine 3000 reagent (Invitrogen, Carlsbad, CA, USA), and siRNA was synthesized by General Biology (Anhui, China) (Table 4). The siRNA was transfected into MG63 and HOS cells according to the manufacturer’s instructions, and the cells were grown stably in MEM containing 10% FBS for 48 h. RNA was extracted from cultured cells using the TRIzol reagent (Invitrogen) and reverse transcribed into cDNA using the reverse transcription premix produced by Vazyme (Nanjing, China). The ChamQ Universal SYBR qPCR Master Mix (Vazyme) and 7500 Fluorescent Quantitative PCR Instrument (Applied Biosystems™, USA) were used to perform real-time quantitative PCR to examine the knockdown efficiency of samples (Table 5). Glyceraldehyde-3-phosphate dehydrogenase (*GAPDH*) (General Biology) was used as a housekeeping gene. The relative mRNA expression was calculated based on the ddCt values, and 2^ddCt represented the fold-change.

**Table 4:** Sequence of siRNA and negative control

Primer	Sequence (5′–3′)
*ARHGAP44* (human) siRNA	GAGAUAGAGUUCAACAUUATT UAAUGUUGAACUCUAUCUCTT
Negative control	UUCUCCGAACGUGUCACGUTT ACGUGACACGUUCGGAGAATT

**Table 5:** Sequence of the PCR primer

Primer	Forward primer (5′–3′)	Reverse primer (5′–3′)
*ARHGAP44*	CACAGCACGCACAAGAAGC	CCCAGGATAGCTGACCCCT
GAPDH	GGAGCGAGATCCCTCCAAAAT	GGCTGTTGTCATACTTCTCATGG

### CCK-8 assay

Cells were transfected, homogeneously inoculated into 96-well plates, and placed in a CO_2_ incubator for growth. Ten microliters of the CCK-8 reagent (GlpBio, Montclair, CA, USA) were added to each well at 24, 48, 72, and 96 h. The incubator was allowed to stand for 3 h, and the OD value at 450 nm was then measured in each well using a microplate reader (BioTek, USA).

### Clone formation assay

The transfected cells were inoculated into six-well plates at a cell density of 1000 cells/well and placed in a CO_2_ incubator for 3 weeks. Culture was discontinued when most of the single-cloned cells in the wells were greater than 50. The cells were washed with PBS, fixed with 4% paraformaldehyde for 30 min, stained with crystal violet staining solution for 10 min, washed again with PBS, dried, and photographed. The number of single-cloned cells greater than 50 was counted.

### Transwell migration assay

A cell suspension (200 μL of cell suspension containing 5 × 10^5^ cells, MEM without FBS) was added to the upper chamber of transwell plates (BioFil, Guangzhou, China). The pore size of transwells was 8.0 μm, and the lower chamber was supplemented with MEM containing 10% FBS. The cells were fixed with 4% paraformaldehyde for 30 min and stained with crystal violet staining solution for 10 min after 24 h incubation. The upper chamber was washed with PBS and observed under a microscope. Five fields of view were photographed in each well, and the cells were counted.

### Wound healing assay

After siRNA transfection, the upper medium was removed and the 200 μL sterile gun head was used perpendicular to the pore plate for marking, washed with PBS, and photographed immediately (0 h). Samples were then cultured in serum-free medium for 24 h and photographed again to detect the migration ability of osteosarcoma cells after siRNA transfection.

### Flow cytometry assay

The transfected cells were inoculated in six-well plates and digested with EDTA-free pancreatic enzyme at 70% confluence. The cells were further centrifuged (2000 rpm, 5 min) to prepare a suspension. Precooled PBS was mixed with 500 μL binding buffer (Seven Biotech, Beijing, China) and 5 μL Annexin V-FITC (Seven Biotech) at 24 °C. The cells were added with 5 μL PI (Seven Biotech) and incubated away from light for 5–15 min. Apoptosis was then detected using flow cytometry. The apoptosis rate was the sum of the percentage of P2-Q2 and P2-Q3 quadrants.

### Western blot analysis

The cells were lysed using RIPA lysis buffer (Epizyme, Shanghai, China) containing 1% protease inhibitor PMSF (Epizyme) on ice for 30 min and centrifuged at 10,000 rpm for 15 min. The supernatant was separated using 10% SDS–PAGE (Epizyme) and transferred onto a PVDF membrane (Biosharp, Guangzhou, China). Thirty micrograms of protein was loaded on the SDS-page of the NC group and the si*ARHGAP44* group. After sealing, the PVDF membrane was incubated with *ARHGAP44* (1:1500; Atlas Antibodies), *p53* (1:1000; Cell Signaling Technology, Danvers, MA, USA), *C-myc* (1:1000; Abcam, Cambridge, UK), *Cyclin D1* (1:1000; Abcam) antibodies, GAPDH (1:1000, Abcam) and β-Actin (1:1000, Abcam). These antibodies were co-incubated overnight at 4 °C. Rapid blocking buffer (Seven Biotech) was used to block the antibodies. After washing the PVDF membranes with TBS-Tween-20 three times, the secondary antibody (Elabscience, Wuhan, China) was added and incubated at 25 °C for 1 h. Next, the indicator proteins were detected using a hypersensitive ECL chemiluminescence kit (Beyotime, Shanghai, China).

### Statistical analysis

The data obtained were expressed as the mean ± standard deviation and statistically analyzed using SPSS 25.0 (IBM Corp. Released 2017. IBM SPSS Statistics for Windows, Version 25.0. Armonk, NY: IBM Corp). In addition, graphs were produced using GraphPad Prism 8 software. One-way analysis of variance (ANOVA) and an unpaired t-test were performed using GraphPad Prism version 8.0.2 for Windows (GraphPad Software, Boston, MA, USA; www.graphpad.com). The Chi-square test was used to compare differences in the staining of osteosarcoma tissue specimens. The log-rank test was used to determine the significance of differences between survival curves, and Spearman's correlation coefficient was used to analyze the correlation between target genes and potential targets. Comparisons between groups were performed using an unpaired t-test, and one-way ANOVA was used for one-way multigroup comparisons. Each group of experiments was repeated at least three times, and *p* < 0.05 indicated statistical significance.

## Results

### *ARHGAP44* expression is elevated in osteosarcoma and correlates with poor prognosis

**Fig. 1:**
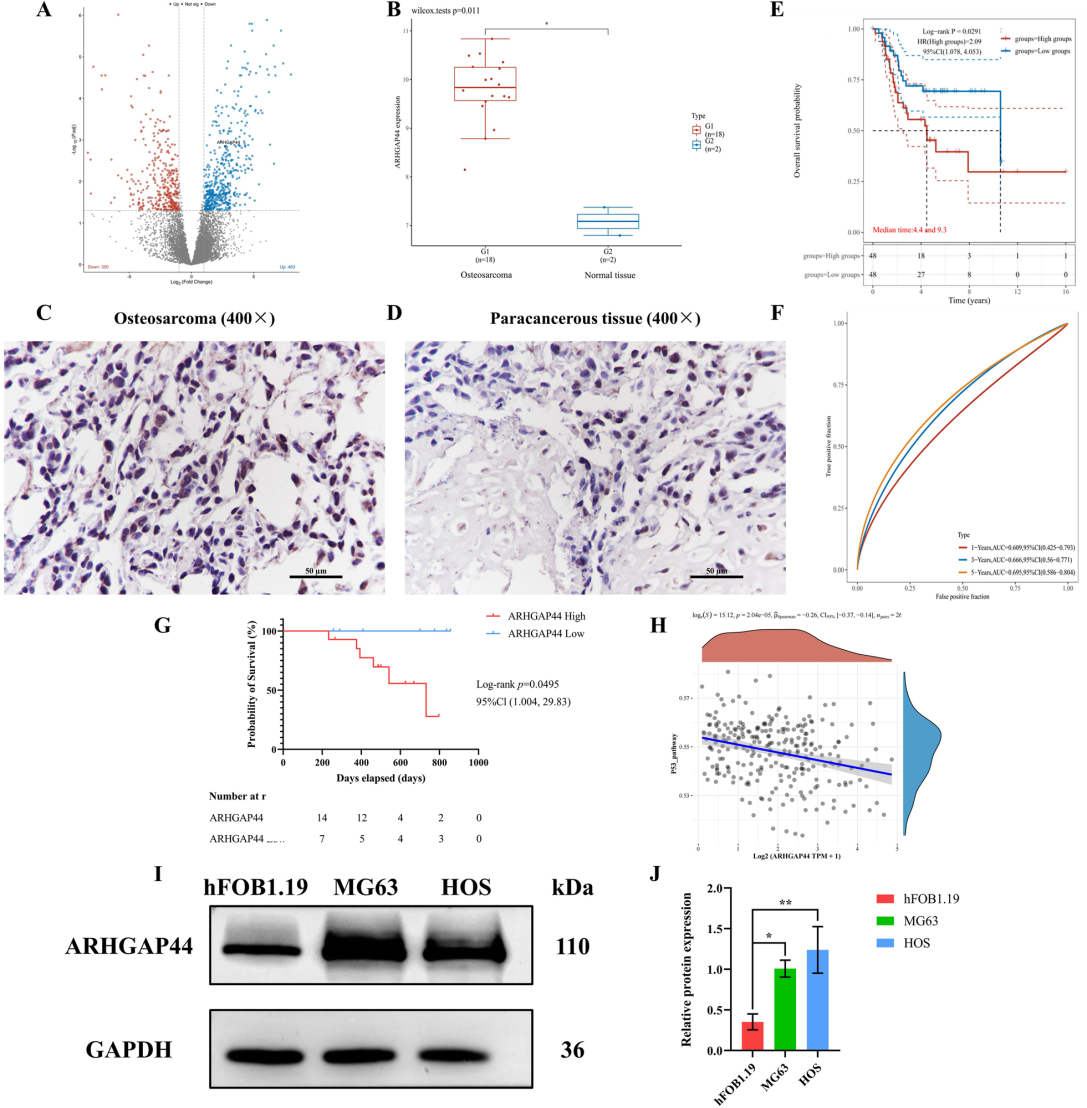
Bioinformatic and immunohistochemical results for the detection of the differential expression of *ARHGAP44* in osteosarcoma, correlation with tumor prognosis, and correlation with *p53*. **A** Differential gene analysis of GSE14359, as obtained using GEO database; **B** The expression level of *ARHGAP44* in osteosarcoma tissues was higher than that in paracancerous tissues, *p* = 0.011; **C** Immunohistochemical results of *ARHGAP44* in osteosarcoma tissue showing deeply stained brown–yellow nuclei, images were recorded using ×400 magnification; **D** Immunohistochemical positive expression of *ARHGAP44* in paracancerous tissues was lower than that in tumor tissues, images were recorded using ×400 magnification; E: Kaplan–Meier survival curves of patients with high and low *ARHGAP44*-expressing osteosarcoma, *p* = 0.0291; F: ROC model for 1-, 3-, and 5-year survival in patients with high *ARHGAP44* expression; **G** KM survival analysis established based on immunohistochemical results of clinical specimens, *p* = 0.0495; **H** TCGA database showing that *ARHGAP44* expression in osteosarcoma was negatively correlated with *p53*, *p* = 2.04e−05, with a Spearman correlation coefficient of − 0.26; I–J: Western blotting results of *ARHGAP44* expression in osteosarcoma and osteoblastic cell lines, * for *p* < 0.05, ** for *p* < 0.01

The GEO database analysis (GSE14359) showed that the expression level of *ARHGAP44* in osteosarcoma was significantly higher than that in normal tissues (*p* = 0.011) (Fig. 1A, B). In addition, the immunohistochemistry results of osteosarcoma tumor specimens and paracancerous tissues showed that the expression of *ARHGAP44* was higher in osteosarcoma tissues than in paracancerous tissues (Fig. 1C, D, Table 6). This gene had an expression rate of 66.7% and 19.0% in osteosarcoma and paracancerous tissues, respectively (*p* < 0.05). Western blotting (Fig. 1I-J) showed that *ARHGAP44* expression in MG63 and HOS osteosarcoma cells was higher than that in hFOB1.19 osteoblastic cells (*p* < 0.05).

**Table 6:** The expression of *ARHGAP44* in osteosarcoma tissues

Group	Number of cases (n)	Positive (n)	Negative (n)	Positive rate (%)	^*χ*2^	*p*
Osteosarcoma tissues	21	14	7	66.7	9.882	0.002
Paracancerous tissues	21	3	18	19.0		

KM survival analysis (GSE39058) indicated that *ARHGAP44* expression in osteosarcoma was associated with prognosis (Fig. 1E, F). The median survival times of patients with osteosarcoma exhibiting high and low expression of *ARHGAP44* were 4.4 and 9.3 years, respectively, and the difference was statistically significant (*p* = 0.0291). Similarly, KM survival analysis (Fig. 1G) based on clinical specimen data showed that high *ARHGAP44* expression was associated with tumor prognosis (*p* = 0.0495). Subsequent analysis based on the TCGA database further revealed that the expression of *ARHGAP44* in osteosarcoma was negatively correlated with the expression of *p53*, with a correlation coefficient of − 0.26. These results suggest that *ARHGAP44* may influence the development of osteosarcoma by negatively regulating the oncogene *p53* (Fig. 1H).

### Relationship between *ARHGAP44* and the malignant biological behavior of osteosarcoma cells

#### Effects of *ARHGAP44* on the proliferation of osteosarcoma cells

**Fig. 2:**
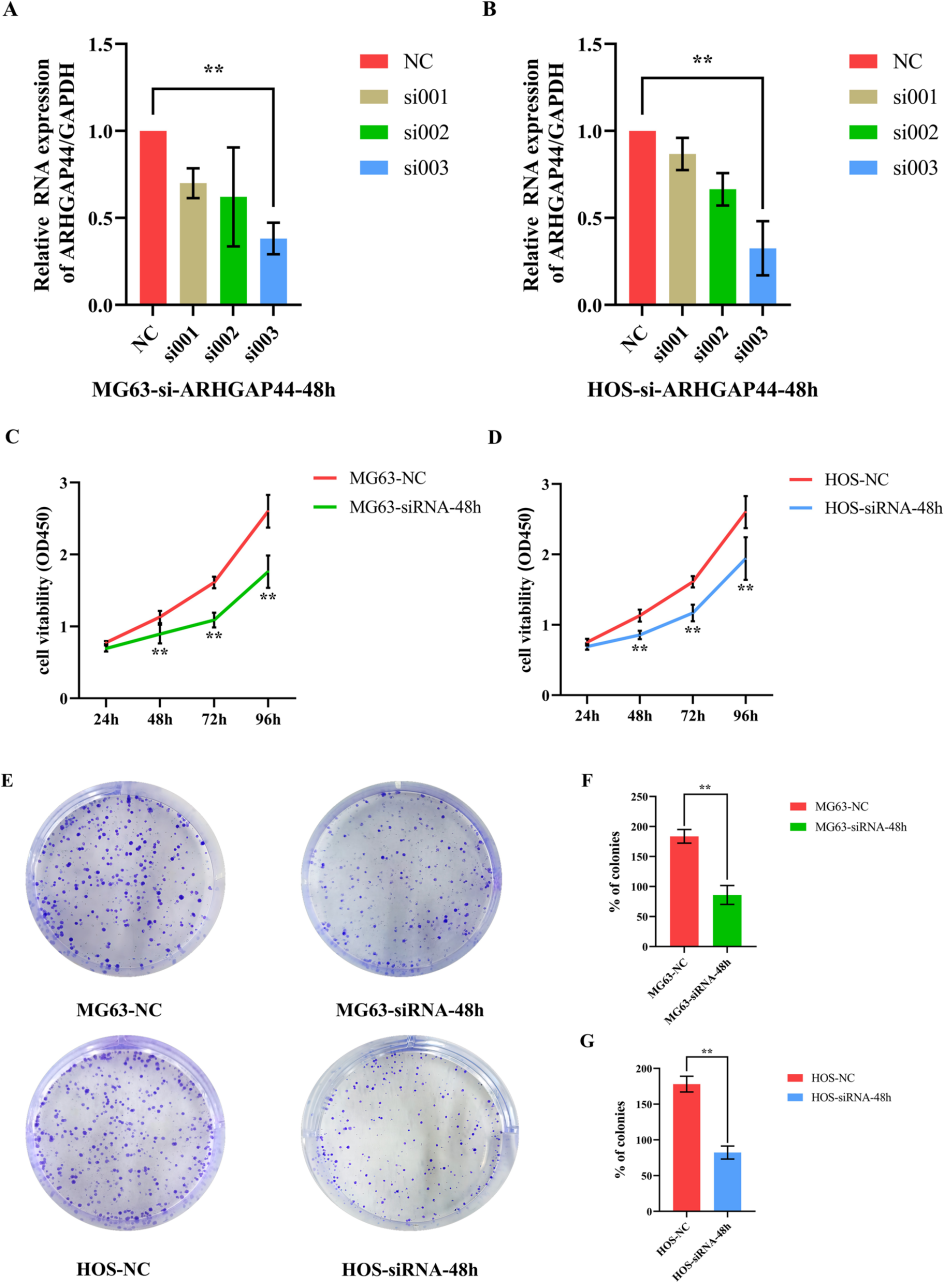
Knockdown cell model of *ARHGAP44* was constructed and the effect of *ARHGAP44* on the proliferative ability of MG63 and HOS cell lines was detected using CCK-8 and clonal formation assays. **A**, **B** Transfection efficiency was verified using qRT − PCR after siRNA transfection, ** for *p* < 0.01; **C**, **D**: Effect of *ARHGAP44* expression inhibition on the cell activity of MG63 and HOS cell lines at 24, 48, 72, and 96 h, ** for *p* < 0.01; **E** Results of clone formation assay of MG63 and HOS cell lines after *ARHGAP44*-siRNA transfection; **F**, **G** Number of clonogenic cells in the MG63 and HOS control group was higher than that in the *ARHGAP44* knockdown group, ** for *p* < 0.01

To investigate the specific role of *ARHGAP44* after clarifying its expression level in osteosarcoma, three *ARHGAP44* knockdown siRNAs were constructed and validated for efficiency. The siRNA with the highest knockdown efficiency (si003) was selected to treat the osteosarcoma cell lines MG63 and HOS (Fig. 2A, B). After successfully constructing the *ARHGAP44* knockdown cell model, we first detected the proliferative ability of the cells using the CCK-8 assay (Fig. 2C, D). When the expression of *ARHGAP44* was downregulated, the proliferative ability of the MG63 and HOS cell lines was significantly weakened compared with that of the control group and showed a time-dependent change (*p* < 0.01). Consistent with the CCK-8 assay results, the clone formation assay suggested that the proliferative capacity of MG63 and HOS cells was significantly reduced after *ARHGAP44* knockdown, and the difference was statistically significant (*p* < 0.01) (Fig. 2E–G).

#### Effects of *ARHGAP44* on the invasion of osteosarcoma cells

**Fig. 3:**
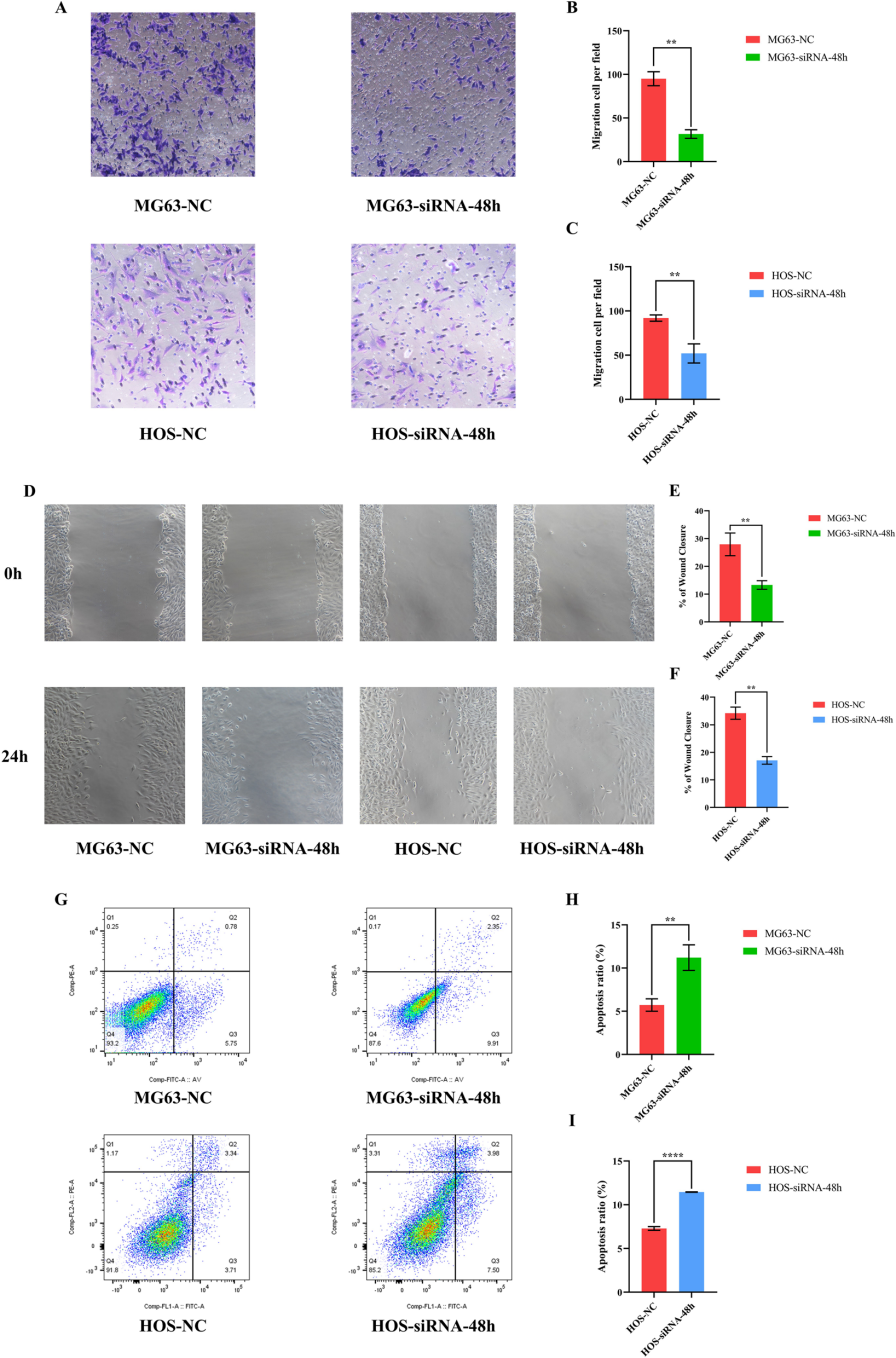
Effects of *ARHGAP44* on the invasion, migration, and apoptosis ability of MG63 and HOS cells, as detected using transwell invasion, wound healing, and flow cytometry assays. **A** Transwell invasion assay results for MG63 and HOS cells transfected with *ARHGAP44*-siRNA; **B** **C** The number of penetrating cells in the MG63 and HOS control groups was significantly higher than that in the *ARHGAP44* knockdown group, ** for* p* < 0.01; **D** Experimental results of the control and *ARHGAP44* knockdown groups at 0 h versus 24 h; **E**, **F** The healing area of the MG63 and HOS control group was larger than that of the *ARHGAP44* knockdown group, ** for* p* < 0.01. **G** Flow cytometry assay results of the *ARHGAP44* knockdown group and the control group; **H**, **I** The apoptosis ratio of MG63 and HOS control groups was significantly higher than that in the *ARHGAP44* knockdown group, ** for* p* < 0.01, **** for* p* < 0.0001

The invasive ability of the cells was also assessed using a transwell invasion assay. The invasive ability of MG63 and HOS cells was significantly inhibited by the downregulation of *ARHGAP44* expression (*p* < 0.01; Fig. 3A–C). This suggests that *ARHGAP44* affects osteosarcoma cell invasion.

#### Effects of *ARHGAP44* expression on the migration of osteosarcoma cells

We examined the migration ability of MG63 and HOS cells using a wound healing assay. When the expression of *ARHGAP44* was downregulated, the migratory ability of MG63 and HOS osteosarcoma cell lines was significantly attenuated compared with the control group (*p* < 0.01) (Fig. 3D–F).

#### Effects of *ARHGAP44* on the apoptosis of osteosarcoma cells

We examined the apoptosis ability of MG63 and HOS cells using a flow cytometry assay. When the expression of *ARHGAP44* was downregulated, the total number of apoptotic cells in both cell lines was significantly increased (*p* < 0.01) (Fig. 3G–I).

### *ARHGAP44* exerts anti-tumor effects through the *p53*/*C-myc*/*Cyclin D1* pathway

**Fig. 4:**
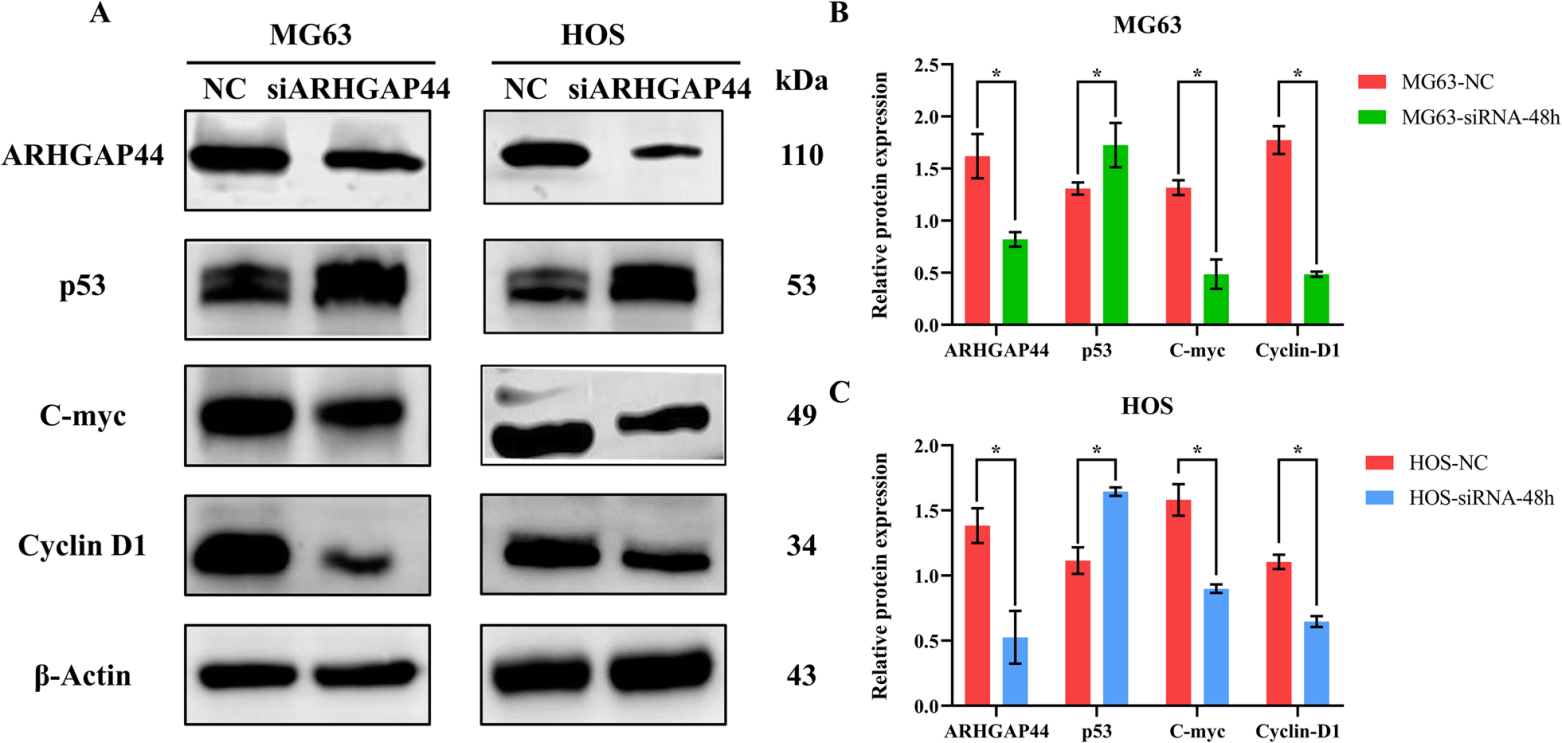
Western blotting was used to detect the protein expression of *p53*, *C-myc*, and *Cyclin D1* after *ARHGAP44* knockdown in osteosarcoma. **A**–**C** The expression of *C-myc* and *Cyclin D1* in the *ARHGAP44* knockdown group was lower than that in the control group, whereas the expression of *p53* was higher than that in the control group. β-Actin served as an internal control, * for *p* < 0.05

To further investigate the specific mechanism of action of *ARHGAP44* in osteosarcoma, the protein expression levels of several molecular pathways were examined using western blotting. When *ARHGAP44* expression was downregulated, the expression levels of *p53* in the MG63 and HOS osteosarcoma cells were significantly higher than those in the control group, whereas the expression of *C-myc* and *Cyclin D1* was significantly lower (*p* < 0.05; Fig. 4A–C). This suggests that the downregulation of *ARHGAP44* expression may play an antitumor role in osteosarcoma by regulating the *p53*/*C-myc*/*Cyclin D1* pathway.

## Discussion

Osteosarcoma is the most common primary bone malignancy in children and adolescents and has a high propensity for local infiltration and metastasis [14]. The combination of surgical resection and adjuvant chemotherapy has led to improved survival in patients with primary osteosarcoma in recent years. However, the prognosis of metastatic or recurrent osteosarcoma remains poor [15]. Therefore, new treatment modalities are required. Modern medical technology and molecular biology have facilitated the rapid development of targeted anticancer drugs. In addition, the study of the pathogenic mechanism in osteosarcoma is of great significance for understanding the development of osteosarcoma and developing new therapeutic targets. Thus, targeted therapies have markedly broad research and application prospects in osteosarcoma.

*ARHGAP44* is a member of the *RhoGAP* family, and loss of *RhoGAP* activity often leads to uncontrolled GTPase activity, which subsequently affects tumor progression [16]. Most *RhoGAP* family members have anticancer effects. However, the biological mechanism of *ARHGAP44* remains controversial [16]. In this study, we first analyzed differences in the expression of *ARHGAP44* using the GEO database. *ARHGAP44* expression in osteosarcoma tissues was significantly higher than that in normal tissues. Immunohistochemical staining of 21 clinically collected osteosarcoma tissue specimens and western blotting results demonstrated high *ARHGAP44* expression in osteosarcoma tissues. In addition, KM survival analysis using both bioinformatic analysis and clinical specimens collected suggested that high *ARHGAP44* expression was negatively correlated with the prognosis of osteosarcoma. This suggests that *ARHGAP44* may play a pro-carcinogenic role in osteosarcoma and may be used as a clinical marker for osteosarcoma prognosis.

Members of the *RhoGAP* family mediate the malignant behavior of tumor cells through *E-cadherin* synergy, *p53* acetylation, and modulation of the *Hippo* and *RhoA*/*AKT* pathways [17–20]. In the present study, we investigated the effects of the *RhoGAP* family member *ARHGAP44* on the malignant biological behavior of osteosarcoma cells. The CCK-8 and clone formation assays demonstrated that the downregulation of *ARHGAP44* expression attenuated the proliferation of MG63 and HOS cells. Results of the transwell invasion and wound healing assays suggested that the downregulation of *ARHGAP44* expression could inhibit the invasion and migration of MG63 and HOS osteosarcoma cells. Furthermore, the downregulation of *ARHGAP44* expression increased the apoptosis of MG63 and HOS cells. These results suggest that *ARHGAP44* plays a key role in the development and progression of osteosarcoma. In addition, *ARHGAP44* may be associated with *p53* mutations that mediate tumor progression [11].

As a downstream target of *RhoGAP* family members, *p53* binds to specific DNA response elements to mediate tumor suppression processes such as cell cycle arrest, DNA repair, apoptosis, and ferroptosis [18, 21]. Inactivation or transformation of *p53* into mutant *p53* can lead to tumor development through downstream pathways such as *MAPK*/*MK2*, *EGFR*/*ERK*, and *C-myc*/*Cyclin D1* [22–24] pathways. Bykov et al. [25] used the small-molecule compound PRIMA-1 to increase *p53* activity, thereby enhancing its cancer inhibitory function [25]. *P53* can play a key role in the pathogenesis of osteosarcoma [26]. In this study, we analyzed the potentially relevant tumor signaling pathways of *ARHGAP44* using the TCGA database. We found a negative correlation between *ARHGAP44* and *p53*. This suggests that *ARHGAP44* influences tumor development in osteosarcoma by regulating *p53* and related molecular pathways, which affects tumor development. Western blot analysis indicated that the downregulation of *ARHGAP44* expression level may lead to the mutation of *p53* and increase its activity, which plays an antitumor role in osteosarcoma, consistent with the findings of previous studies [22, 25]. This suggests that *ARHGAP44* regulates *p53* in osteosarcoma, further revealing the upstream regulatory mechanisms of this gene.

**Fig. 5:**
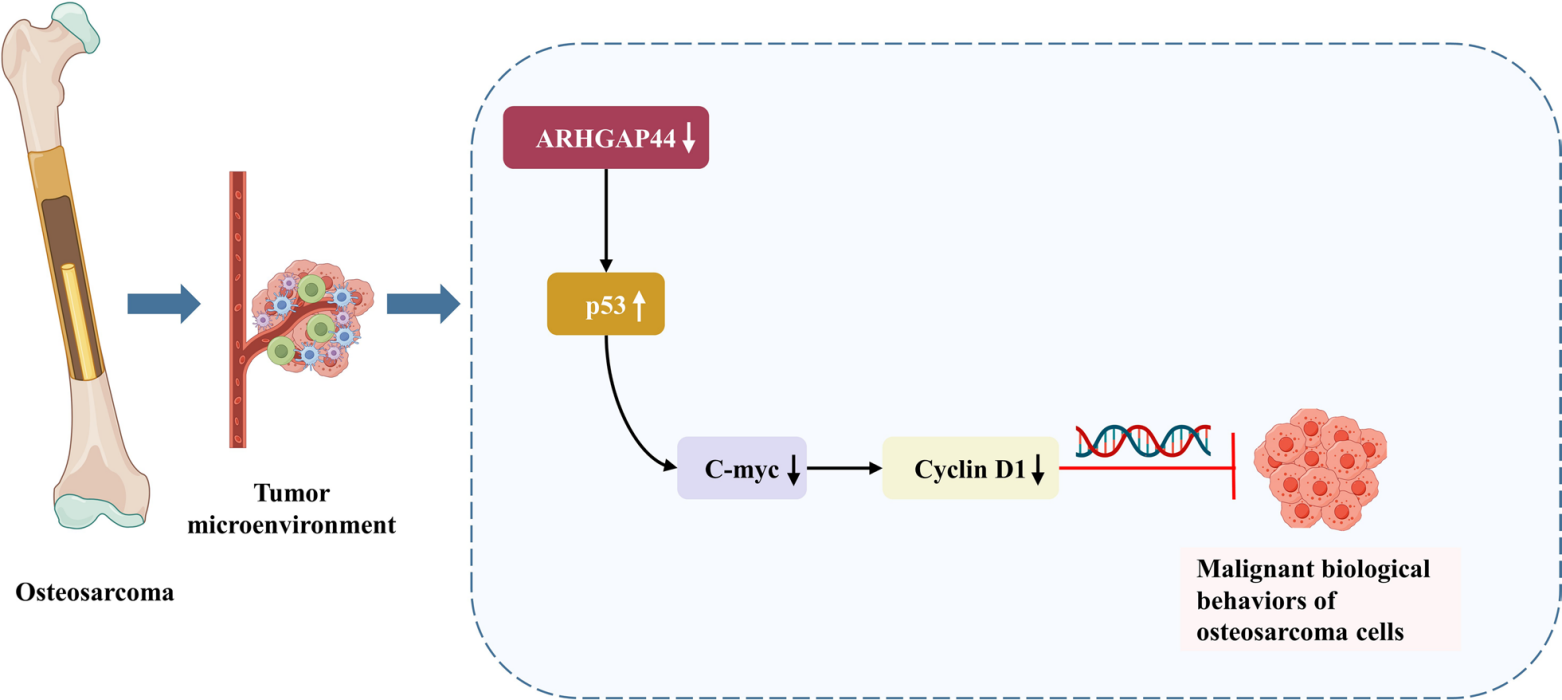
Mechanism of the downregulation of *ARHGAP44* expression to inhibit malignant biological behaviors through the *p53*/*C-myc*/*Cyclin D1* pathway in osteosarcoma

*C-myc* and *Cyclin D1* are classical molecular signaling pathways that have been widely studied in malignant tumors such as breast cancer and lung cancer [27]. *C-myc* is highly expressed as a proto-oncogene in breast, rectal, and non-small cell lung cancers and plays a role in malignant progression [28, 29]. Similarly, *Cyclin D1* is overexpressed in most malignant tumors and is associated with a poor prognosis [30]. In osteosarcoma, the *C-myc*/*Cyclin D1* pathway often contributes to osteosarcoma progression by altering the cell cycle, DNA repair, and cell migration [31, 32]. There is also a regulatory relationship between *p53*, *C-myc*, and *Cyclin D1*. Oyang et al. [33] found that *LPLUNC1* reduces the proliferation of nasopharyngeal carcinoma tumor cells through the *p53*/*C-myc* pathway. In breast cancer, metformin reduces the expression of *Cyclin D1* by upregulating the *p53* expression, thereby promoting tumor apoptosis [34]. However, the roles of the *ARHGAP44*/*p53* and *C-Myc*/*Cyclin D1* pathways in osteosarcoma remain unclear. The present study revealed that *C-myc* and *Cyclin D1* may be inhibited by the downregulation of *ARHGAP44* and upregulation of *p53* expression levels, which is consistent with the findings of Oyang and Yenmis et al. [33, 34]. These results suggest that the downregulation of *ARHGAP44* expression may inhibit the proliferation, invasion, and migration of osteosarcoma tumor cells through the *p53*/*C-myc*/*Cyclin D1* pathway (Fig. 5).

A limitation of this study is that the direct regulatory relationship between *p53*, *C-myc*, and *Cyclin D1* was not further verified. However, these limitations will be further supplemented and improved in subsequent studies.

The present study demonstrates, for the first time, that the downregulation of *ARHGAP44* expression may inhibit osteosarcoma development by regulating the *p53*/*C-myc*/*Cyclin D1* pathway. These findings provide new insights into the molecular pathogenesis of osteosarcoma. These findings further suggest that *ARHGAP44* may serve as a prognostic biomarker and potential therapeutic target for osteosarcoma in clinical practice.

## Data Availability

The datasets supporting the conclusions of this study are included in the article.
