# Correction: ARHGAP44-mediated regulation of the p53/C-myc/Cyclin D1 pathway in modulating the malignant biological behavior of osteosarcoma cells

**DOI:** 10.1186/s13018-023-04454-5

**Published:** 2023-12-18

**Authors:** Shizhe Li, Jiancheng Xue, He Zhang, Guanning Shang

**Affiliations:** 1 Department of Orthopedics, The First Affiliated Hospital of Jinzhou Medical University, Jinzhou, Liaoning China; 2 Department of Orthopedics, Shengjing Hospital of China Medical University, Shenyang, Liaoning China; 3 Medical Research Center, Shengjing Hospital of China Medical University, Shenyang, Liaoning China

**Correction: Journal of Orthopaedic Surgery and Research (2023) 18:910** 10.1186/s13018-023-04406-z

Following publication of the original article [1], the authors identified an error in Fig. 1. The correct figure is given below.

**Fig. 1:**
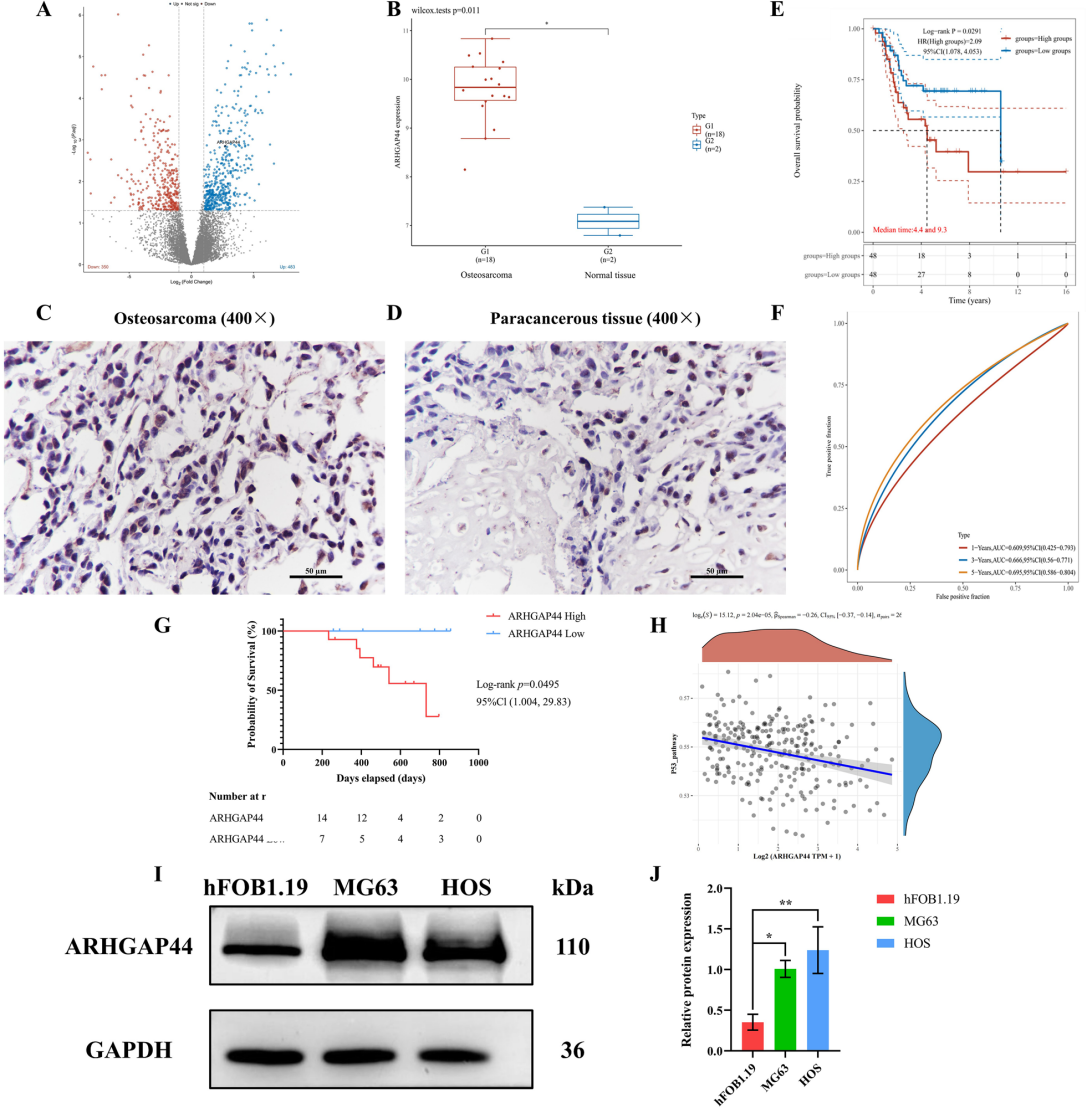
Bioinformatic and immunohistochemical results for the detection of the differential expression of *ARHGAP44* in osteosarcoma, correlation with tumor prognosis, and correlation with p53. **A** Differential gene analysis of GSE14359, as obtained using GEO database; **B** The expression level of *ARHGAP44* in osteosarcoma tissues was higher than that in paracancerous tissues, *p* = 0.011; **C** Immunohistochemical results of *ARHGAP44* in osteosarcoma tissue showing deeply stained brown–yellow nuclei, images were recorded using ×400 magnification; **D** Immunohistochemical positive expression of *ARHGAP44* in paracancerous tissues was lower than that in tumor tissues, images were recorded using ×400 magnification; **E** Kaplan–Meier survival curves of patients with high and low *ARHGAP44*-expressing osteosarcoma, *p* = 0.0291; **F** ROC model for 1-, 3-, and 5-year survival in patients with high *ARHGAP44* expression; **G** KM survival analysis established based on immunohistochemical results of clinical specimens, *p* = 0.0495; **H** TCGA database showing that *ARHGAP44* expression in osteosarcoma was negatively correlated with *p53*, *p* = 2.04e−05, with a Spearman correlation coefficient of −0.26; **I**–**J** Western blotting results of *ARHGAP44* expression in osteosarcoma and osteoblastic cell lines, * for *p* < 0.05, **for *p* < 0.01

The original article [1] has been corrected.

